# ExGenNet: Learning to Generate Robotic Facial Expression Using Facial Expression Recognition

**DOI:** 10.3389/frobt.2021.730317

**Published:** 2022-01-04

**Authors:** Niyati Rawal, Dorothea Koert, Cigdem Turan, Kristian Kersting, Jan Peters, Ruth Stock-Homburg

**Affiliations:** ^1^ Chair for Marketing and Human Resource Management, Department of Law and Economics, Technical University of Darmstadt, Darmstadt, Germany; ^2^ Intelligent Autonomous Systems, Department of Computer Science, Technical University of Darmstadt, Darmstadt, Germany; ^3^ AI and Machine Learning Group, Department of Computer Science, Technical University of Darmstadt, Darmstadt, Germany; ^4^ Centre for Cognitive Science and Hessian Center for AI (hessian.AI), Darmstadt, Germany; ^5^ Leap in Time—Work Life Research Institute, Darmstadt, Germany

**Keywords:** facial expression generation, humanoid robots, facial expression recognition, neural networks, gradient descent

## Abstract

The ability of a robot to generate appropriate facial expressions is a key aspect of perceived sociability in human-robot interaction. Yet many existing approaches rely on the use of a set of fixed, preprogrammed joint configurations for expression generation. Automating this process provides potential advantages to scale better to different robot types and various expressions. To this end, we introduce ExGenNet, a novel deep generative approach for facial expressions on humanoid robots. ExGenNets connect a generator network to reconstruct simplified facial images from robot joint configurations with a classifier network for state-of-the-art facial expression recognition. The robots’ joint configurations are optimized for various expressions by backpropagating the loss between the predicted expression and intended expression through the classification network and the generator network. To improve the transfer between human training images and images of different robots, we propose to use extracted features in the classifier as well as in the generator network. Unlike most studies on facial expression generation, ExGenNets can produce multiple configurations for each facial expression and be transferred between robots. Experimental evaluations on two robots with highly human-like faces, Alfie (Furhat Robot) and the android robot Elenoide, show that ExGenNet can successfully generate sets of joint configurations for predefined facial expressions on both robots. This ability of ExGenNet to generate realistic facial expressions was further validated in a pilot study where the majority of human subjects could accurately recognize most of the generated facial expressions on both the robots.

## 1 Introduction

Perceived sociability is an important aspect in human-robot interaction (HRI), and users want robots to behave in a friendly and emotionally intelligent manner (Nicolescu and Mataric, 2001; Hoffman and Breazeal, 2006; Ray et al., 2008; de Graaf et al., 2016). Studies indicate that in any interaction, 7% of the affective information is conveyed through words, 38% is conveyed through tone, and 55% is conveyed through facial expressions (Mehrabian, 1968). This makes facial expressions an indispensable mode of communicating affective information and, subsequently, generating appropriate and realistic facial expressions, which can be perceived by humans, and a key ability for humanoid robots.

While methods for automated facial expression recognition have been a research field in human-robot interaction for several years (see Li and Deng (2020); Canedo and Neves (2019), for overviews), facial expression generation for humanoid robots is a younger line of research (Breazeal, 2003; Kim et al., 2006; Ge et al., 2008; Horii et al., 2016; Meghdari et al., 2016; Silva et al., 2016). Moreover, most of the existing studies use preprogrammed joint configurations, for example, adjusting the servo motors movement for the eyelids and the mouth to form the basic expressions in a hand-coded manner (Breazeal, 2003; Kim et al., 2006; Ge et al., 2008; Bennett and Sabanovic, 2014; Silva et al., 2016). While this allows studying human reactions to robot expressions, it requires hand-tuning for every individual robot and re-programming in case of hardware adaptations on a robot’s face. In contrast, the automated generation of robot facial expressions could alleviate the “hard-coding” of expressions, thereby making it easier to scale to different robots in a more principled manner. Still, there are only a few studies that learn different configurations for expressions automatically (Breazeal et al., 2005; Horii et al., 2016; Churamani et al., 2018), and most of the studies only learn to generate a single configuration for each facial expression. Humans, however, usually exhibit a variety of different expressive ways instead of a single configuration per facial expression. Such high expressiveness can be tedious to hand-tune, which can potentially be achieved easier in a generative setting.

**FIGURE 1:**
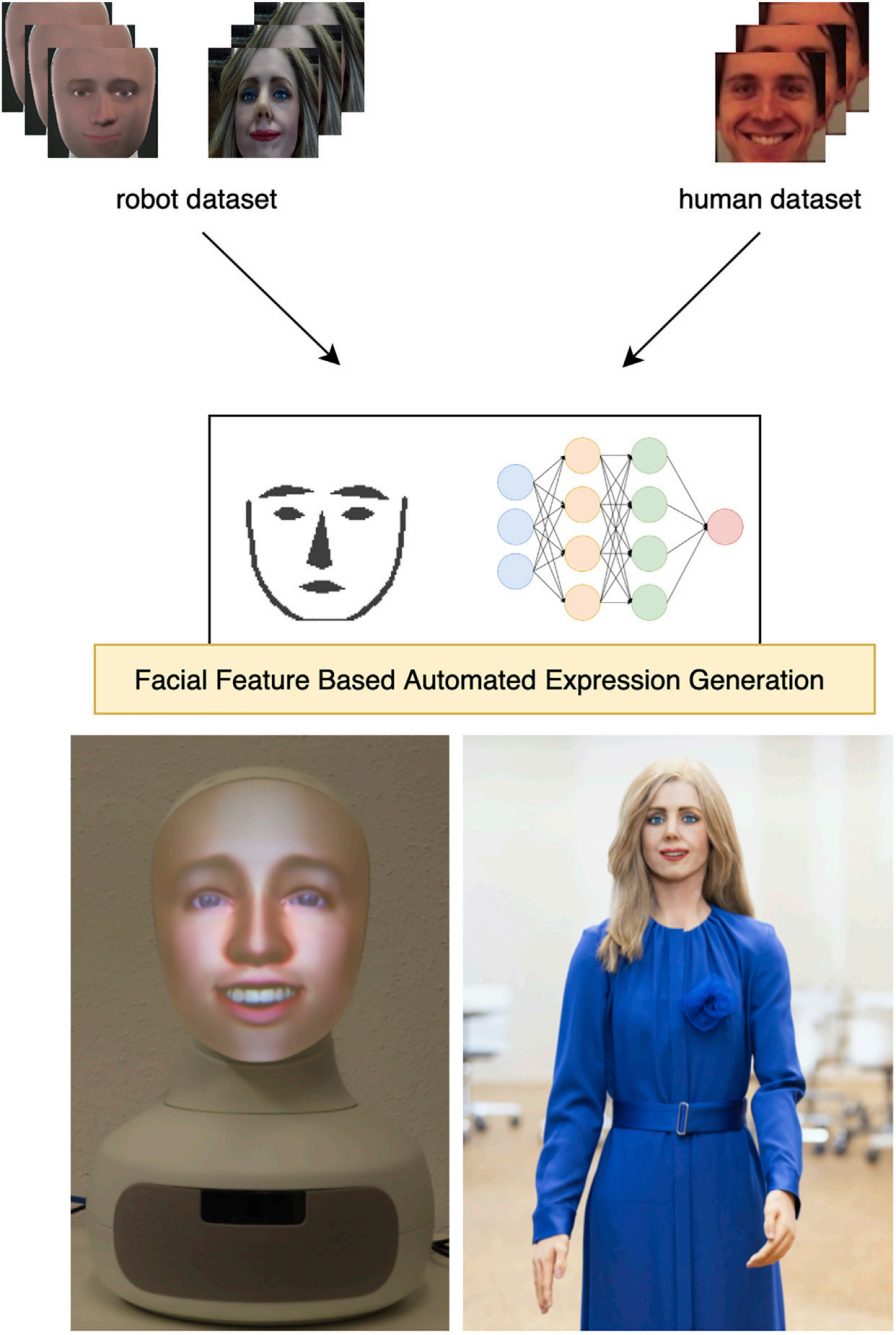
We propose a novel approach to automatically learn multiple joint configurations for facial expressions on highly humanoid robots, such as Alfie **(left)** and Elenoide **(right)**. For generation of multiple facial expressions we utilize a classifier based on simplified images of extracted facial features from a mixed dataset of human and robot training pictures.

In this article, we propose a novel approach to automatically learn multiple joint configurations for facial expressions on humanoid robots, called ExGenNet. In particular, we suggest utilizing state-of-the-art deep network approaches for image-based human facial expression recognition as feedback to train a pipeline of robot facial expression generation (See Figure 1 for an overview). We evaluate ExGenNet on the two robots Elenoide and Alfie (Furhat Robot) with highly human-like faces, where we make use of facial feature extractors for smooth transfer between the different robot types. The learned facial expressions are additionally evaluated in a pilot study where we investigate how images of the robots displaying the autogenerated expressions are perceived by humans.

To summarize, our main contributions are threefold. First, ExGenNet introduces a novel automated way of learning multiple joint configurations per expression via a gradient-based minimization of the loss between the intended expression and the expression predicted by the trained classifier. Second, ExGenNet uses facial features as a shared representation between human training data and different robots. Finally, we present insights on expression generation on highly humanoid robots and the way they are perceived by humans.

The rest of the article is structured as follows. In Section 2, we give a short overview of related work followed by the details of our algorithm in Section 3. In Section 4, we present the results of our experimental evaluations on two robots with highly human-like faces. Finally, we conclude and discuss future work in Section 5.

## 2 Related Work

Facial expression recognition has been well studied in human-robot interaction (HRI) (e.g., Cid et al., 2014; Meghdari et al., 2016; Simul et al., 2016; Bera et al., 2019). As deep learning methods have become popular, facial expression recognition nowadays mostly consists of preprocessing the facial images and directly feeding them into deep networks to predict an output (Li and Deng, 2020). Among all approaches, Convolutional Neural Networks (CNNs) are widely used for performing facial expression recognition during HRI (see Rawal and Stock-Homburg (2021) for an overview). In particular, CNNs are used for end-to-end recognition, i.e., given the input image, the output is directly predicted by the network. ExGenNets employ CNNs for the expression classification part of our pipeline.

In the field of computer vision, there are many works that tackle facial expression generation within pictures of persons, other characters, or even animals. For instance, Noh and Neumann (2006) transferred motion vectors from a source to a target face model. Here, the target face model could have completely different geometric proportions and mesh structures, compared to the source face model. Pighin et al. (2006) created photorealistic 3D facial models from photographs of a human, and further created continuous and realistic transitions between different facial expressions by morphing between different models. Pumarola et al. (2019) implemented a Generative Adversarial Network (GAN) to animate a given image and render novel expressions in a continuum. While these approaches are capable of generating “images” of robots with different facial expressions, they are incapable of directly being transferred to robots since the joint angles need to be optimized to realize an expression.

This may explain why most approaches to facial expression generation on humanoid robots rely on hand-tuned expression generation. Only a few studies so far introduced automated ways for expression generation based on simple Neural Networks (Breazeal et al., 2005), Restricted Boltzmann Machines (RBM) (Horii et al., 2016), and Reinforcement Learning (RL) (Churamani et al., 2018).

Specifically, Breazeal et al. (2005) used neural networks to learn a direct mapping of human facial expressions onto a robot’s joint space. Horii et al. (2016) used an RBM to generate expressions on an iCub robot. The forward sampling in the RBM is to recognize the human counterpart’s expression based on facial, audio, and gestural data during HRI, while the backward sampling is to generate facial, audio, and gestural data for the robot. Churamani et al. (2018) generated expressions on their robot Nico using RL. They used an actor-critic network where the actor network receives the current mood affect vector of the robot and generates an action (LED configurations for the eyebrows and mouth) corresponding to this state. The action and the state are then fed into the critic network that predicts a Q-value for the state-action pair. The robot receives an award based on symmetry in wavelengths generated by the network.

All of these studies only generate single configurations per expression. In contrast, ExGenNets are able to generate a range of joint configurations per expression. Additionally, even though the robots used in related approaches are humanoid robots, their faces are not as human-like as the faces of the robots used in our experimental evaluation. Since these robots may start entering the uncanny valley (Mori et al., 2012), we consider evaluation of the human perception of expressions generated on robots with highly human-like faces is particularly important.

## 3 ExGenNet: Expression Generation Network

**FIGURE 2:**
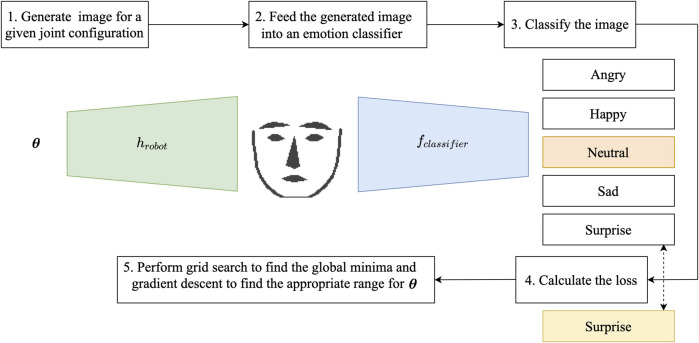
Network structure of the proposed ExGenNet (Expression Generation Network) and optimization procedure of the joint configuration θ . First, the generator network generated an image of facial features for a given joint configuration (θ) . Next, we feed this image into an expression classifier that classifies the image into five categories: angry, happy, neutral, sad, and surprise. The cross-entropy loss between the predicted expression and desired expression is calculated. We find the value of the joint configuration (global minima) for which the cross-entropy loss is the minimum by performing a grid search. To find the local minima (mean and standard deviation), we backpropagate the cross-entropy loss through the classifier and the generator networks to update the joint configuration. The goal is to find the appropriate range of joint configuration for a given facial expression.

Expression Generation Networks (ExGenNets) learn multiple joint configurations for previously defined facial expressions on humanoid robots. To achieve this, they combine a generator network, which reconstructs a simplified image of facial landmarks for a given configuration, together with a CNN-based expression classifier. Figure 2 summarizes the overall approach.

Let us now introduce ExGenNets in detail. In Section 3.1, we present the details of the automated expression generation of facial landmarks. Section 3.2 introduces the facial feature-based expression classifier. Lastly, Section 3.3 explains the optimization of joint values for different expressions.

### 3.1 Simplified Expression Image Generator

**FIGURE 3:**
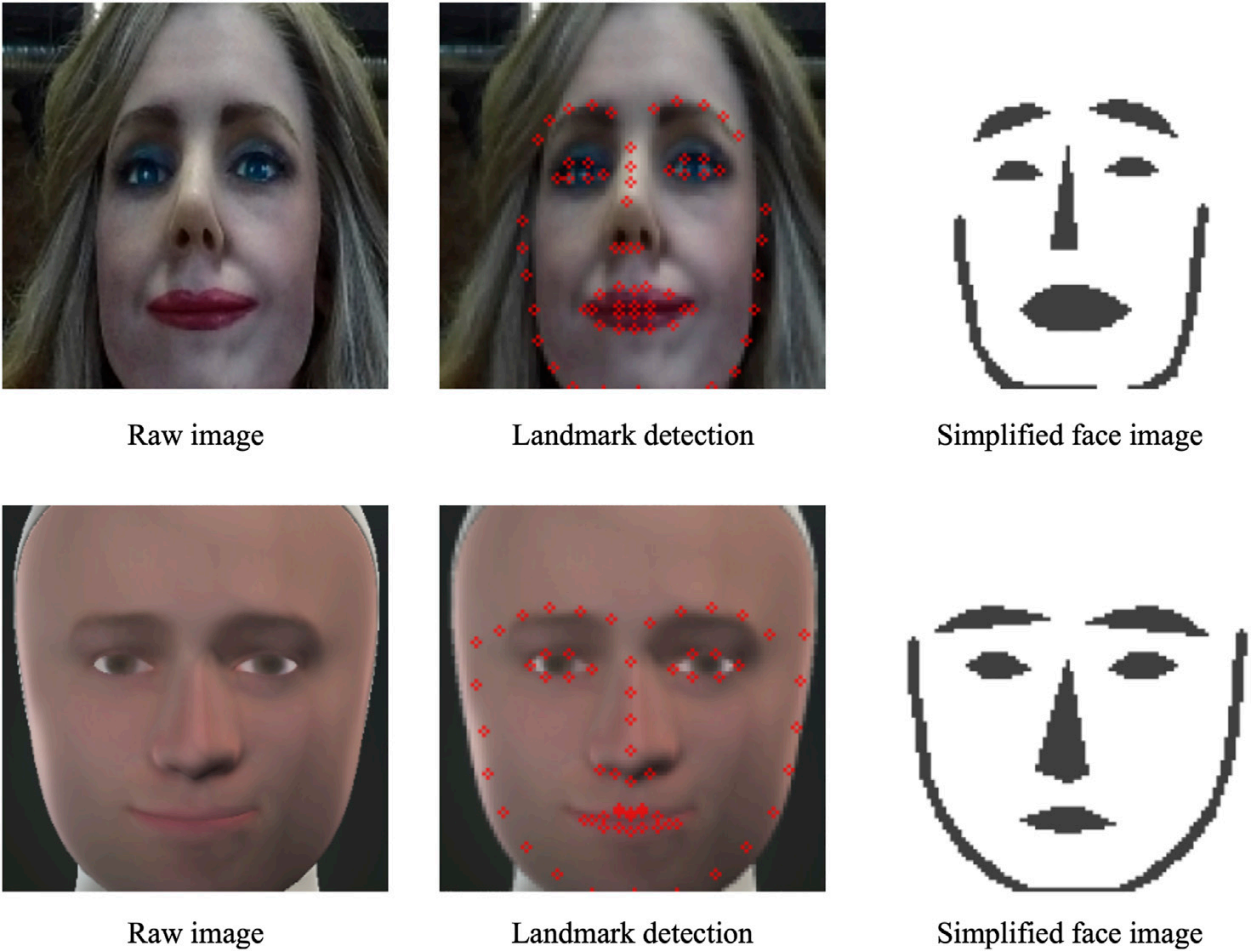
An example of a simplified image showing the facial landmarks in black. The facial landmarks are detected using dlib (King, 2009). The simplified expression image generator generates images of this kind.

To learn a mapping between the robot joint configurations and facial expressions, we train a generator network to convert from joint angles to a simplified facial image

$$X_{\text{image}}=h_{\text{robot}}\left(\theta \right)$$ (1)

where ***X***_image_ denotes the image of the facial features generated by the network, *h*_robot_ the generator network, and θ the joint configuration of the robot. To reduce computational effort, we obtain simplified images by applying the dlib facial feature detector (King, 2009) on the full images of the robot and constructing a smaller and less detailed black and white image from this. An example of such a simplified image can be seen in Figure 3.

For the generator network we use a CNN with five layers, which generates images of size 48 × 48 × 1. The network structure is given in Table 1. We created a dataset of the images of facial feature extraction at various values of joint configurations from the robot. Next, we trained the generator network *h*_robot_ to generate images of facial feature extraction from robot joint configuration. The generator network that we use is similar to the generator network in standard Generative Adversarial Networks (GANs), except we do not use a discriminator to train the images being generated. As the output consists of only simplified images of facial features, we found it is sufficient to train the generator network by reducing the mean squared error between the pixels of the actual output and the predicted output.

**TABLE 1:** CNN Network structure for generator.

Layer (type)	Output shape	Param #
dense (Dense)	(None, 9,216)	55,296/27,648
batch_normalization (BatchNormalization)	(None, 9,216)	36,864
leaky_relu (LeakyReLU)	(None, 9,216)	0
reshape (Reshape)	(None, 6, 6, 256)	0
conv2d_transpose (Conv2DTranspose)	(None, 6, 6, 128)	294,912
batch_normalization_1 (BatchNormalization)	(None, 6, 6, 128)	512
leaky_relu_1 (LeakyReLU)	(None, 6, 6, 128)	0
conv2d_transpose_1 (Conv2DTranspose)	(None, 12, 12, 64)	73,728
batch_normalization_2 (BatchNormalization)	(None, 12, 12, 64)	256
leaky_relu_2 (LeakyReLU)	(None, 12, 12, 64)	0
conv2d_transpose_2 (Conv2DTranspose)	(None, 24, 24, 32)	18,432
batch_normalization_3 (BatchNormalization)	(None, 24, 24, 32)	128
leaky_relu_3 (LeakyReLU)	(None, 24, 24, 32)	0
conv2d_transpose_3 (Conv2DTranspose)	(None, 48, 48, 1)	288

### 3.2 Feature-Based Expression Classifier

Most studies that perform facial expression recognition directly train on facial images (Barros et al., 2015; Ahmed et al., 2019). In contrast, we train the Convolutional Neural Network (CNN) on simplified versions of the training images generated from landmarks detected via dlib (King, 2009). As training images, we used the KDEF dataset (Lundqvist et al., 1998) and additional hand-labeled training images for our robots Alfie and Elenoide. We used data augmentation to slightly translate the training images both vertically and horizontally so that the faces are visible completely. We also evaluate the models trained with different combinations of data, i.e., only human facial expressions, only robot facial expressions, and both combined. The use of the extracted features, i.e. landmark points, makes the classifier hereby less sensitive to environmental changes such as lighting conditions and generalizes better between different robot types and human images. From the set of labeled training images, the classifier should learn the mapping from the simplified facial images to discrete predefined expressions

$$y_{\text{expression}}=f_{\text{classifier}}\left(X_{\text{image}}\right)\in \left\{angry,happy,neutral,sad,surprise\right\}$$ (2)

with the expression classifier *f*_classifier_ and the simplified image of the extracted facial features **X**_image_ of size 48 × 48 × 1. For the expressions classifier, we used CNN with four convolutional layers and one fully-connected layer. The network structure is given in Table 2.

**TABLE 2:** CNN Network structure for expression classifier.

Layer (type)	Output shape	Param #
conv2d (Conv2D)	(None, 46, 46, 32)	320
conv2d_1 (Conv2D)	(None, 44, 44, 64)	18,496
max_pooling2d (MaxPooling2D)	(None, 22, 22, 64)	0
dropout (Dropout)	(None, 22, 22, 64)	0
conv2d_2 (Conv2D)	(None, 20, 20, 128)	73,856
max_pooling2d_1 (MaxPooling2D)	(None, 10, 10, 128)	0
conv2d_3 (Conv2D)	(None, 8, 8, 128)	147,584
max_pooling2d_2 (MaxPooling2D)	(None, 4, 4, 128)	0
dropout_1 (Dropout)	(None, 4, 4, 128)	0
flatten (Flatten)	(None, 2048)	0
dense_1 (Dense)	(None, 1,024)	2,098,176
dropout_2 (Dropout)	(None, 1,024)	0
dense_2 (Dense)	(None, 5)	5,125

### 3.3 Automated Expression Generation

Here, we explain in detail how we automatically calculate and obtain the values of joint configurations θ by performing grid search and gradient descent. To automatically find a set of joint configurations that generate a desired facial expression *y*_*j*_ on the robot, we minimize the cross-entropy loss *L* between the predicted expression of the classifier $\hat{y_{j}}=\left(f_{\text{classifier}}\left(h_{\text{robot}}\left(\theta \right)\right)\right)$ and the desired expression

$$L=-\frac{1}{N}\overset{N}{\underset{j=1}{\sum }}\left(y_{j}\log\hat{y_{j}}\right)+\left(1-y_{j}\right)\log\left(1-\hat{y_{j}}\right)$$ (3)

with *N* predefined facial expressions. To find stable joint configurations, we determined and averaged the loss for five generator networks and five classifier networks.

Specifically, we first find the global minima for θ values in the whole range. To this end, we consider θ values at discrete steps and find the combination of joint angles for which the loss is minimal for each expression. Then, we find the global minima. We assume a range for values of θ instead of just a single joint configuration per expression by considering $\theta ∼N\left(\mu ,\sigma^{2}\right)$ where μ is the mean and $\sigma^{2}$ is the standard deviation. We used reparameterization to sample from μ and σ by considering θ=μ+ϵʘσ and correspondingly propagate the gradients to minimize the loss via gradient descent

$$\min_{\theta }L\left(f_{\text{classifier}}\left(h_{\text{robot}}\left(\theta \right)\right)\right)$$ (4)

using element-wise multiplication ʘ and Gaussian noise ϵ∼N(0,1) .

The mean and the variance are updated by gradient descent

$$\mu_{k+1}=\mu_{k}-\alpha \frac{\partial L}{\partial \mu },\sigma_{k+1}=\sigma_{k}-\alpha \frac{\partial L}{\partial \sigma }$$ (5)

using a learning rate *α* and computing the gradients by ∂L/∂μ=∂L/∂θ and ∂L/∂σ=∂L/∂θʘϵ .

Algorithm 1 summarizes the overall approach. Here, *f* is the expression-classifier and *h* is the image generated for Alfie’s and Elenoide’s face. The loss L and gradients $\partial L/\partial \vec{\theta }$ and $\partial L/\partial \vec{\theta }ʘ\vec{\epsilon }$ are averaged over minibatch.

Algorithm 1 Generating multiple configurations for various expressions.

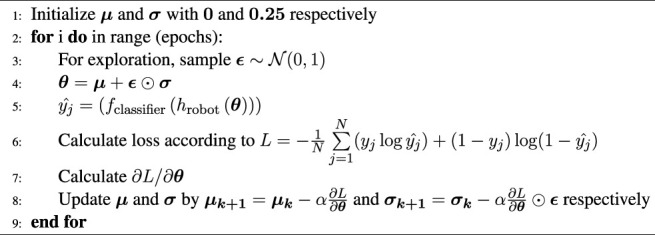


## 4 Experimental Evaluation

**FIGURE 4:**
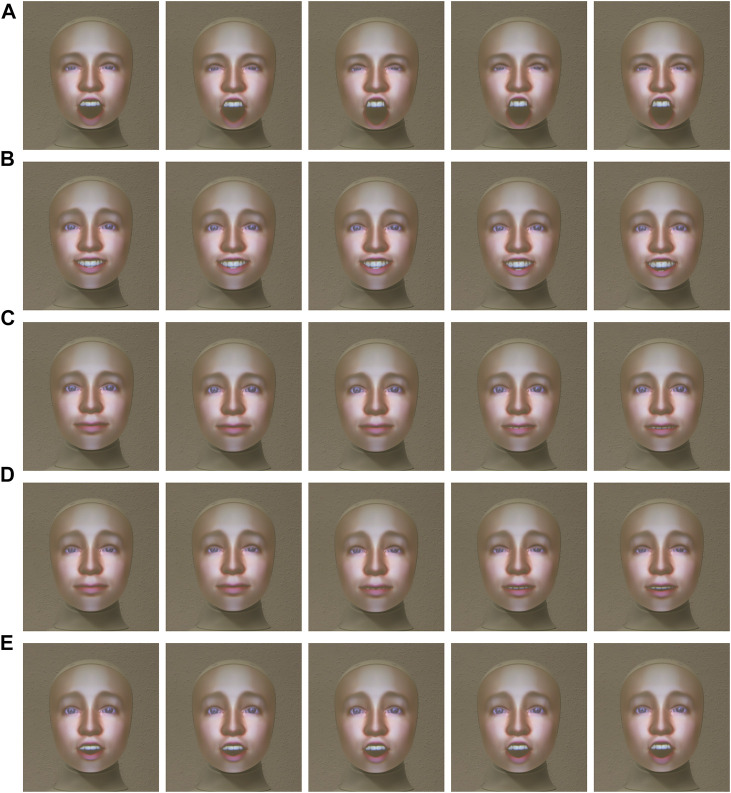
Alfie (real Furhat Robot) displaying **(A)** Angry, **(B)** Happy, **(C)** Neutral, **(D)** Sad, and **(E)** Surprise expressions at μ−2σ,μ−σ,μ,μ+σ,μ+2σ respectively. Here, μ is the mean and σ is the standard deviation. We obtain the μ and the σ values for various expressions from the ExGenNet by reparameterizing θ as θ=μ+ϵʘσ .

**FIGURE 5:**
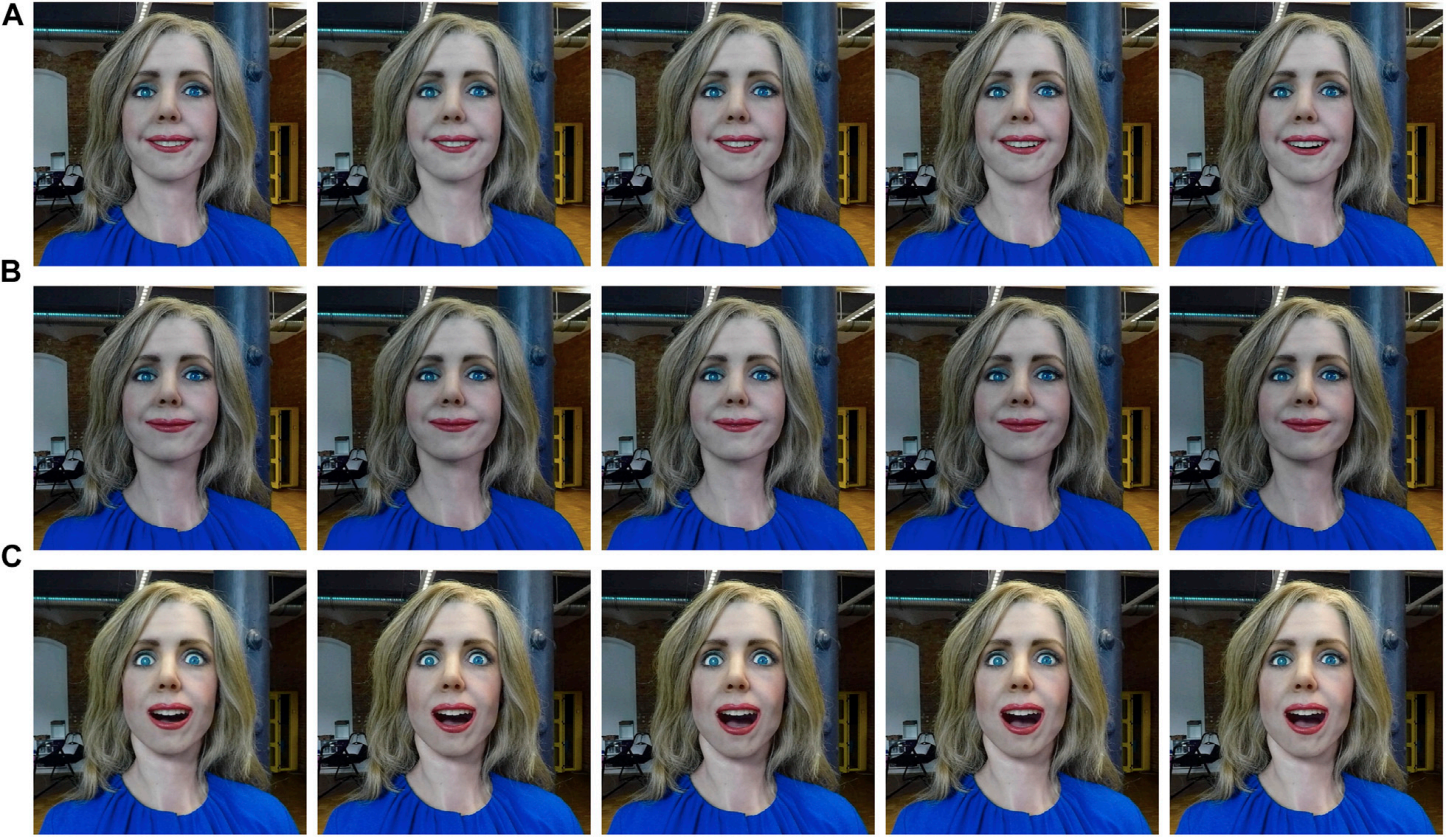
Elenoide displaying **(A)** Happy, **(B)** Neutral, and **(C)** Surprise expressions at μ−2σ,μ−σ,μ,μ+σ,μ+2σ respectively. Here, μ is the mean and σ is the standard deviation. We obtain the μ and the σ values for various expressions from the ExGenNet by reparameterizing θ as θ=μ+ϵʘσ .

We evaluated our proposed method on two highly humanoid robots, named Alfie (Figure 4) and Elenoide (Figure 5). First, we present the results of expression generation using our novel ExGenNet approach in Section 4.1. Additionally, we report in Section 4.2 the results of a pilot study, where we evaluated how the generated expressions are perceived by humans. Finally, we discuss the remaining limitations of our method in Section 4.3.

### 4.1 Expression Generation on the Robots

We conducted experiments with the approach described in Section 3 on our robots Alfie and Elenoide. The code is written in Python using TensorFlow. Adam was used as an optimizer. For the grid search, we considered discrete intervals of 0.1 for each of the three joint angles for Elenoide and 0.2 for each of the six joint angles for Alfie. For fine-tuning the $\vec{\theta }$ values and obtaining a range for each expression, we initialized the mean to 0 and standard deviation to 0.25 for all joint angles $\left(\vec{\theta }\right)$ for both Elenoide and Alfie. We considered the following joints for Alfie: BLINK_LEFT, BLINK_RIGHT, BROW_UP_LEFT, BROW_UP_RIGHT, BROW_DOWN_LEFT, BROW_DOWN_RIGHT, SMILE_OPEN, PHONE_CH_J_SH, and PHONE_BIGAAH. For Elenoide, we considered the joints in the eyes, eyebrows, and mouth (mouth opening and mouth corner pull). Alfie is able to generate angry, happy, neutral, sad, and surprise expressions (see Figure 4). Elenoide is able to generate only happy, neutral, and surprise expressions (see Figure 5). Owing to restrictions in the degrees of freedom in the mouth and eyebrows, Elenoide is not able to generate negative facial expressions like sad and angry.

**FIGURE 6:**
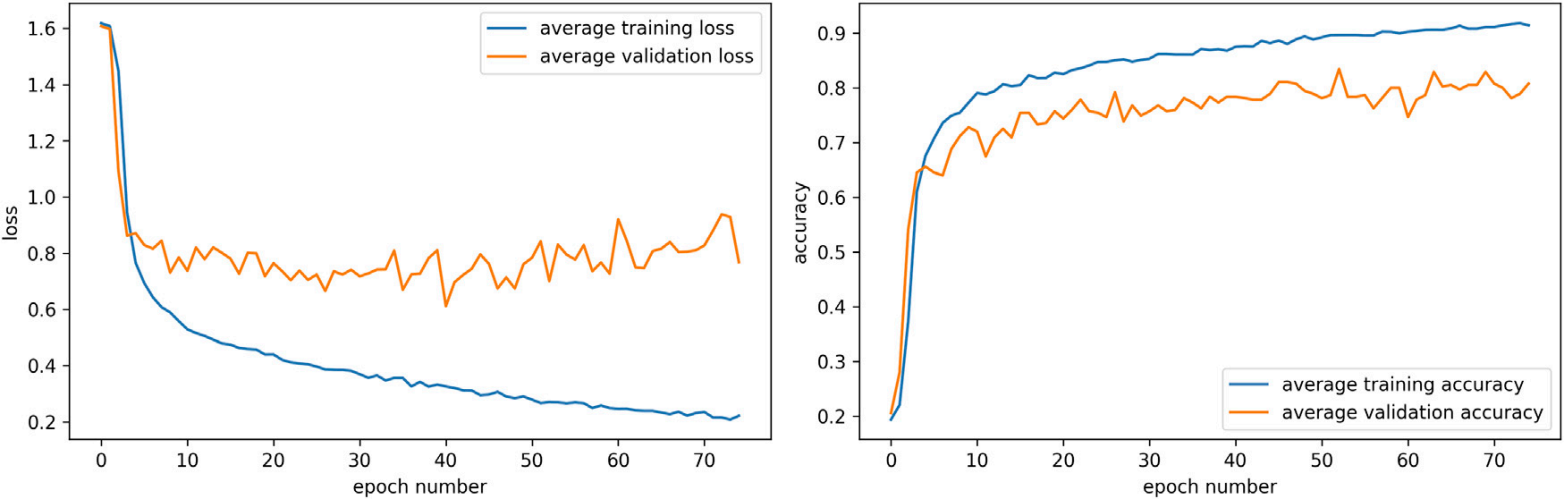
Graphs showing the average loss and the average accuracy for the five classifier models trained with random seeds. The epoch number where the loss was minimum was selected for obtaining the values of the joint angles for each of the five classifier networks.

To have consistent values for the joint configurations, we averaged the loss in Equation 4 for five generator and five classifier models with different random initializations. The graphs showing the loss and accuracy for the five classifier models can be seen in Figure 6. The epoch number where the loss is minimum is selected for the five classifier models that are then used in the ExGenNet for obtaining the values of the joint angles for various expressions.

**FIGURE 7:**
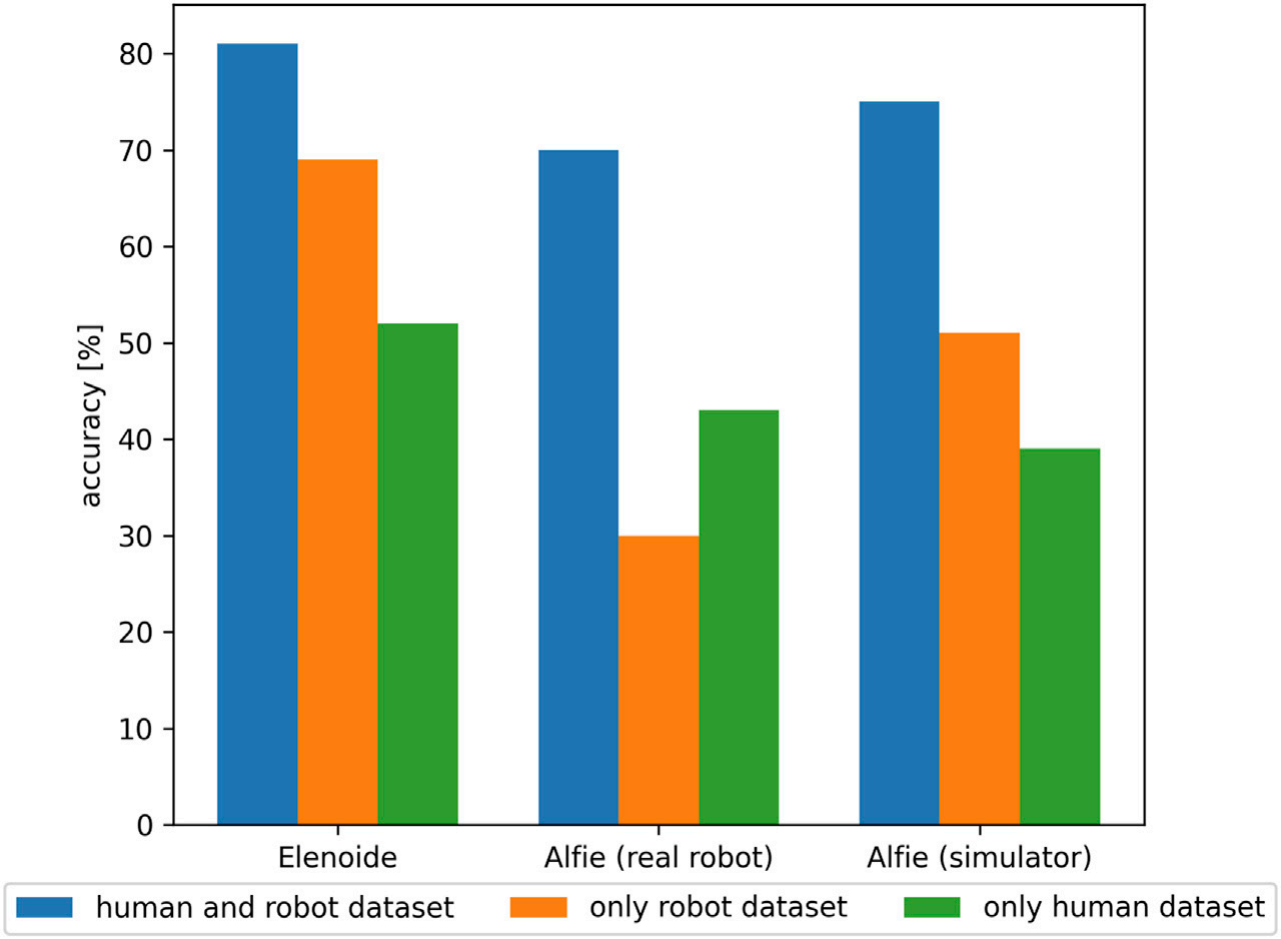
Comparison of classifier accuracy for Elenoide, Alfie (real robot), and Alfie’s simulator when trained with combined human and robot dataset (blue), only robot dataset (orange), and only human dataset (green). Classifiers trained with combined human and robot dataset performed the best for all Elenoide, Alfie, and Alfie’s simulator.

After generating the expressions for the two robots, we verified the facial expressions using the previously trained classifiers. For both Elenoide and Alfie, we tested the five classifiers to recognize the expressions for images obtained at μ−2σ,μ−σ,μ,μ+σ,μ+2σ . We also sample 10 images from μ and σ and average the classification results. On Elenoide, the average accuracy for recognizing the expressions correctly was 81% for the five classifiers trained on human and robot dataset (see Figure 7). For classifiers trained on only robot and only human datasets, the average accuracies were 69 and 52% respectively. In the case of Alfie, we trained the ExGenNet and obtained the mean and standard deviation for various expressions on the simulator. Figure 4 shows the final results on the real robots. For the classification results on the images of the real robot, the average accuracy was 70% for the five classifiers trained on the human and robot dataset. The classifiers trained on only human and only robot datasets had average accuracies of 43 and 30%, respectively. We also tested the classification results on Alfie’s simulator. The average accuracy for the five classifiers trained on human and robot data combined was 75%, followed by the classifiers trained on only robot data 51%. The accuracy for the classifiers trained on only human data was 39%. For Elenoide, Alfie, and Alfie’s simulator, the classifier that is trained on the combined dataset of humans and robots gives the best results. While the classifier trained on only robot data has a higher expression recognition rate for Elenoide and Alfie’s simulator, the classifier trained on only human data has better results in the case of Alfie. This is because the classifier trained on only robot data consists of simplified face images of only Elenoide and Alfie’s simulator. As Alfie and Alfie’s simulator has a different rendering and the classifier trained on only robot data overfits to the data of Elenoide and Alfie’s simulator, the classifier trained on only robot data performs worse than the classifier trained on human data for Alfie.

### 4.2 Human Perception of Generated Expressions

To validate the results further, we also conduct a pilot study to investigate how images of autogenerated expressions are perceived by humans. Here, we conducted three surveys, one for humans to be able to recognize the expressions generated on Elenoide (happy, neutral, and surprise) and another two for humans to be able to recognize the positive expressions (happy, neutral, and surprise) and the negative expressions (angry, neutral, and sad) on Alfie. In each survey, we randomly showed three images per expression and asked two questions. The first question is how positive or negative the expressions look and the second question is to recognize the expression in the image out of five categories: angry, happy, neutral, sad, and surprise. There is also an additional optional category for “other” expressions in case it seems that the expression in the image does not belong to any of the five categories. The first question is rated between 1 and 7, where 1 indicates that the expression looks negative and 7 indicates that the expression looks positive. The second question is rated on a five-point Likert scale, where 1 indicates strongly disagree and 5 indicates strongly agree. In between the survey, there was a screening question to check the participants’ attention. The screening question asked the participants to choose “strongly agree” on the same five-point Likert scale.

We obtained responses from 30 participants for each survey. We did not consider the responses of the participants who answered the screening question incorrectly and ended up evaluating the results for 27 participants in the case of Elenoide, 29 participants to recognize the positive expressions for Alfie, and 27 participants to recognize the negative expressions for Alfie. Out of all the participants, 65% were male and 35% were female with an average age of 35 years. We performed the Pearson’s correlation test using the Scipy library in Python. First, the mean values of all the expressions for all participants on a five-point Likert scale were calculated. Next, the intended labels were assigned. For example, if the intended class is happy, the intended labels would be [1,5,1,1,1,1]. The Pearson’s correlation test was performed to compare the classes chosen by humans with the intended classes. The Scipy library in Python returns both the Pearson’s correlation coefficient and a two-tailed *p*-value for the Pearson’s function. The Pearson’s correlation coefficient and the two-tailed *p*-value are given in Table 3 for various expressions.

**TABLE 3:** Results of Pearson’s correlation test for various expressions on Elenoide and Alfie.

Robot	Expression	Pearson’s correlation coefficient	*p*-value
Alfie	Happy	0.87	p < 0.001
	Neutral	0.68	p < 0.001
	Surprise	0.82	p < 0.001
Alfie	Angry	0.37	0.04
	Neutral	0.45	0.01
	Sad	−0.17	0.37
Elenoide	Happy	0.63	p < 0.001
	Neutral	0.24	0.19
	Surprise	0.89	p < 0.001

We found that in the case of Alfie, all the positive expressions are positively correlated, happy being the most positively correlated, followed by surprise and thereafter neutral. In the case of negative expressions, sad was found to be negatively correlated. Alfie does not have a joint configuration in its mouth that can make the corners of its mouth turn downward in case of a sad expression, always forming a slight smile. Therefore, Alfie cannot express sadness the way a human does. Neutral and angry were found to be positively correlated. The *p*-value for all expressions except sad was less than 0.05. Therefore, the correlation coefficient for all expressions except sad is significant. In the case of Elenoide, all three expressions were positively correlated, “surprise” is the most positively correlated, followed by happy and neutral. For neutral, the *p*-value was greater than 0.05, implying that the result is not significant. While observing the data obtained from the participants, it was found that the neutral images of Elenoide were reported as both happy and neutral. This is probably because the corners of the mouth for Elenoide are always pulled upward even for neutral expressions, forming a smile.

### 4.3 Discussion of Limitations

While our proposed approach successfully generated multiple joint configurations for facial expressions of the two robots, which were correctly recognized by human subjects in the majority of cases, we also noticed some limitations of the current method.

The robots used in our experiments seemed to show hardware limitations, which made it harder to generate negative expressions than positive ones. The mouth corners of both the robots cannot directly move down, making it hard for the two robots to express sadness the way humans did in the training images. Also in the case of the neutral expression on Elenoide, human subjects would sometimes mistake it for happy, due to the constantly slightly upward pull of the mouth corners. One question here is whether we require robots to show negative expressions and if yes, whether they should show these in the same way humans would or not. Besides considering full ranges for expression generation possibilities in hardware design, it might also be interesting to investigate if there would be ways to express sad on our robots, which would deviate from the human training images but could still be classified correctly by human subjects.

Another limitation of our method in the current form is that even though we are able to generate multiple joint configurations per expression, they do not directly map to a level of intensity of the expression. However, humans usually are able to generate and recognize different intensities in expressions and we, therefore, consider this an interesting direction for extending our method in future work.

## 5 Conclusion and Future Work

We introduce a novel framework, called ExGenNet, to optimize facial expressions for the robots Alfie and Elenoide. This deep generative approach is based on neural networks for recognizing expressions. Using our ExGenNet, we obtained a range of joint configurations for Alfie to be able to express angry, happy, neutral, sad, and surprise and for Elenoide to express happy, neutral, and surprise. The limitations in the degrees of freedom in the mouth and eyebrows of Elenoide prevent ExGenNet from being able to generate negative expressions like sad and angry. In the pilot study, humans were asked to recognize the facial expressions generated by the two robots. The Pearson’s correlation test showed that while angry, happy, neutral, and surprise are positively correlated, sad is negatively correlated in the case of Alfie. In the case of Elenoide, all three expressions surprise, happy, and neutral are positively correlated. However, the result for neutral in the case of Elenoide was not significant. In future work, one should conduct human-robot interaction (HRI) experiments where the robots are able to recognize the human facial expressions and generate their own facial expressions accordingly. One should also extend facial expressions to other modalities such as gesture-based expressions in the case of Elenoide.

## Data Availability

Publicly available datasets were analyzed in this study. This data can be found here: https://kdef.se/download-2/index.html.
